# 340. GeneXpert Ultra for Diagnosis of Central Nervous System Tuberculosis in the High Tuberculosis Prevalence Country - A Prospective Study from India

**DOI:** 10.1093/ofid/ofac492.418

**Published:** 2022-12-15

**Authors:** Santhanam Naguthevar, Deepak Kumar, Shivang Sharma, D M ID, Gopal Krishna Bohra, Durga Shankar Meena, Naresh Kumar Midha, Sarika P Kombade, Vidhi Jain, M K Garg, Kuldeep Singh

**Affiliations:** AIIMS JODHPUR, Jodhpur, Rajasthan, India; AIIMS jodhpur, Jodhpur, Rajasthan, India; AIIMS, Jodhpur, Rajasthan, India; AIIMS, Jodhpur, Rajasthan, India; AIIMS, Jodhpur, Rajasthan, India; AIIMS, Jodhpur, Rajasthan, India; AIIMS, Jodhpur, Rajasthan, India; AIIMS, Jodhpur, Rajasthan, India; AIIMS, Jodhpur, Rajasthan, India; AIIMS, Jodhpur, Rajasthan, India

## Abstract

**Background:**

Early diagnosis and initiation of antitubercular treatment are vital steps to reducing the impact of TB in high burden countries. The molecular method like GeneXpert has significantly improved the diagnosis of TB. GeneXpert Ultra is more sensitive to the diagnosis and helps in paucibacillary samples like cerebrospinal fluid (CSF). This study aims to provide its utility in diagnosis of central nervous system tuberculosis (CNS TB) in high TB burden geography.

**Methods:**

This prospective observational study included the suspected cases of CNS TB with age ≥12 years. Patients who presented with ≥ 5 days history of CNS symptoms suggestive of tuberculosis with a lancet score of ≥ 6 on combination of clinical, CSF and imaging criteria were included in the study. Patients diagnosed with aetiology other than tuberculosis for CNS disease were excluded. CSF was analysed for GeneXpert Ultra and mycobacterial culture. The sensitivity, specificity, positive predictive value (PPV), and Negative predictive value (NPV) of GeneXpert Ultra were analysed in comparison with culture results.

**Results:**

A total of 107 patients with mean age of 33.2±18.5 years were analysed. CSF mycobacterial culture was positive in 45.8% (n=49) of patients. However, *Mycobacterial tuberculosis* via GeneXpert Ultra was detected in 57.9% (n=62) of patients. Sensitivity, specificity, PPV and NPV for GeneXpert Ultra were found 83.7%, 63.8%, 20.4% and 97.2% respectively. According to the lancet score, 70.1% (n=75) and 29.9% (n=32) of patients had probable and possible CNS TB, respectively. Among them, definitive CNS TB were diagnosed in 65.3% (n=49) and 65.6% (n=21) patients with probable and possible CNS TB, respectively.

**Conclusion:**

GeneXpert Ultra has an excellent sensitivity and negative predictive value for the diagnosis of CNS TB in CSF samples. Even with possible CNS TB according to the lancet score, it is better to consider antitubercular treatment in a high TB burden country.

**Disclosures:**

**All Authors**: No reported disclosures.

